# Associations between neighborhood walkability, convenience stores, and cognitive decline among Black adults

**DOI:** 10.1093/geroni/igaf122.3714

**Published:** 2025-12-31

**Authors:** Ketlyne Sol, Melissa Lamar, Ana Capuano, Lisa Barnes

**Affiliations:** University of Miami, Boca Raton, Florida, United States; Rush University Medical Center, Chicago, Illinois, United States; University of Chicago, Chicago, Illinois, United States; Rush University Medical Center, Chicago, Illinois, United States

## Abstract

Neighborhood walkability facilitates life sustaining activity such as obtaining nutrition, which is vital for cognitive health, but mechanisms are unknown. Convenience stores within the neighborhood may provide a source of nutrition if options are limited. The current study tested whether existence of convenience stores explained the relationship between neighborhood walkability and cognitive decline in Black older adults. Participants (N = 786; M-age=73.4; 79% female) were drawn from two prospective community-based aging studies in Chicago in which participants were administered a battery of cognitive tests assessing five cognitive domains from which a global composite was computed. Participant addresses were geocoded and linked to walkability scores and sum of convenience stores within 0.5 miles of the participant’s home. Multilevel regression analyses adjusting for demographics, health factors, and neighborhood SES examined whether the association between neighborhood walkability and cognitive trajectories was explained by convenience stores. More walkability was associated with better baseline visuospatial ability (B=.0015, SE=.0008, p=.0683), but faster decline (B=-.0002, SE=.0001, p=.0139). When adding convenience stores to the fully adjusted models, the effect of walkability on visuospatial ability became slightly stronger for the baseline effect (B=.0022, SE=.0009, p=.0219), while the longitudinal effect was unchanged (B=-.0002, SE=.0001, p=.0183). Walkability was not associated with any other cognitive domains at baseline or longitudinally. Although walkability may have a selective effect on decline in visuospatial abilities, these findings do not support convenience stores as a potential mechanism underlying neighborhood walkability and cognitive trajectories. Future studies are needed to examine other potential mechanisms linking walkability to change in cognition.

